# Influence of Cu on the sustainable synthesis and thermoelectric properties of the half-Heusler TiNiSn†

**DOI:** 10.1039/d4cc06695b

**Published:** 2025-04-04

**Authors:** Blair F. Kennedy, Aaron B. Naden, Clemens Ritter, Jan-Willem G. Bos

**Affiliations:** a EaStCHEM School of Chemistry, University of St Andrews North Haugh, St Andrews KY16 9ST UK j.w.g.bos@st-andrews.ac.uk; b Institut Laue-Langevin 71 avenue des Martyrs, CS 20156 38042 Grenoble Cedex 9 France

## Abstract

Doping of TiNiSn with abundant Cu is highly beneficial for its thermoelectric performance. *In situ* neutron powder diffraction reveals that the half-Heusler formation reaction is complete within 3–4 h. Using these insights we optimise the synthesis of Cu doped TiNiSn from powder precursors, minimising decomposition, and achieve a high *zT* = 0.9 at 723 K.

## Abstract 2

Findings from *in situ* neutron diffraction are used to reduce processing times, yielding TiNiCu_0.03_Sn samples with *zT* = 0.9 from powder precursors.

## Graphical abstract

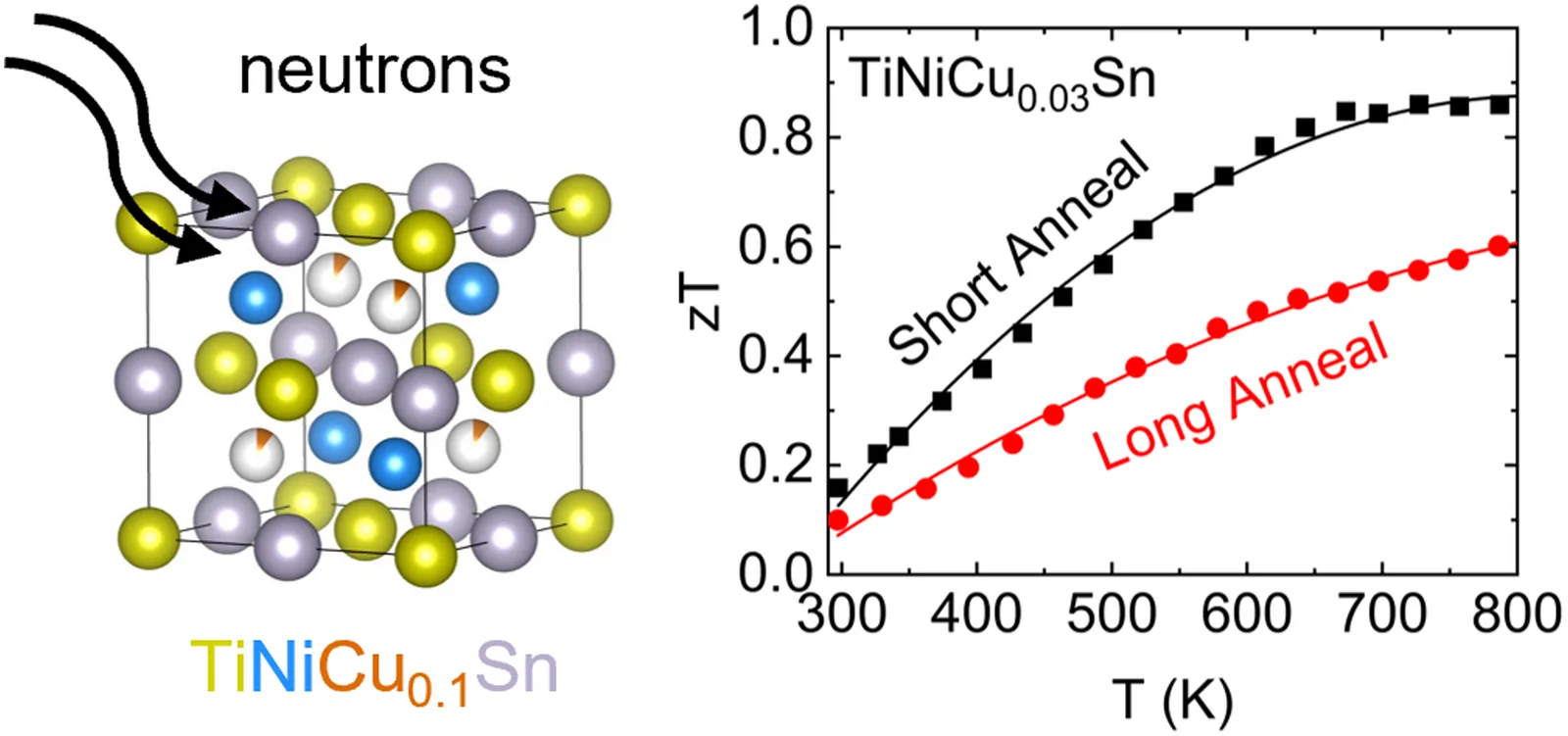

The use of thermoelectricity, where a temperature gradient can generate electricity, for power generation has gained increased interest as demand for renewable energy rises. Despite this potential global application, the actual implementation of thermoelectricity has been limited by high costs and poor understanding on how to upscale lab-based synthesis routes to industrial capacities.^1,2^

The performance of any individual thermoelectric (TE) material is quantified by a figure of merit *zT* = (*S*^2^/*ρκ*)*T*. Here, *S* is the Seebeck coefficient, *ρ* is the electrical resistivity, and *κ* is the sum of the lattice (*κ*_L_) and electronic (*κ*_el_) thermal conductivity, with *T* the absolute temperature.^3^

For mid to high temperature applications (473–873 K), one of the most exciting TE materials currently investigated are the intermetallic half-Heusler (HH) alloys.^4,5^ Of these, the XNiSn (X = Ti, Zr, Hf) system is the premier n-type material. To achieve high *zT* values, the inclusion of Hf (to reduce *κ*_L_) and Sb (for carrier doping) has been widely employed.^6^ However, the scarcity and price of these elements is suboptimal for large scale application. Currently, TiNiSn-based materials, which contain only abundant elements and thus are the most desirable n-type compositions, are of rapidly increasing interest for more large scale application.^4^

Recently, an alternative to alloying with Zr/Hf and doping with Sb has emerged.^7–11^ This uses abundant Cu, which occupies the normally empty interstitial 4d site. Introduction of 0.03 Cu is sufficient to achieve, *zT* = 0.9 at 773 K in TiNiSn prepared by arc melting, hot pressing and annealing.^11^ The Cu serves a dual role, improving the electronic properties, by a combined doping and a band engineering effect, and by reducing *κ*_L_ through strong phonon scattering.^11^ Introduction of Cu also improves sample homogenisation, greatly aiding the mixing of Ti, Zr and Hf and improving grain growth, in samples prepared using a powder-based synthesis route.^8^

In this work, we aim to establish if (1) the presence of Cu leads to faster phase formation of TiNiSn when prepared from elemental powders and (2) if high quality TE samples can be obtained using a powder-based synthesis route, avoiding melt-based processing, but without extensive annealing.

To gain insight into the impact of Cu, the formation of a TiNiCu_0.1_Sn sample was studied by *in situ* neutron powder diffraction (NPD). This composition has more Cu than desirable for good TE performance but enables its location by diffraction. To understand if long duration annealing is essential, beneficial or even detrimental, optimally doped TiNiCu_0.03_Sn was annealed for 24 h and 168 h, followed by characterisation of the structure, microstructure and TE properties.

*In situ* monitoring of the formation of undoped TiNiSn has been recently reported, with two major melting events found to be crucial for phase formation.^12^ First at ∼230 °C the melting of Sn leads to its reaction with Ni, forming Ni_3_Sn_4_ and Ni_3_Sn_2_ up to 750 °C. Second, the melting of Ni_3_Sn_4_ at 750–800 °C leads to the sudden formation of full-Heusler (FH) TiNi_2_Sn (major) and HH TiNiSn (minor), alongside small amounts of Ti–Ni binaries. During annealing (>800 °C, 900 °C was used) TiNiSn gradually becomes the major phase. Notably, only ∼7 h annealing was needed to complete the reaction in this *in situ* study, compared to the common weeklong timescales often used in laboratory synthesis.

The same heating regime as the published study was used for TiNiCu_0.1_Sn (3 °C min^−1^ to 900 °C, holding at that temperature). The collected NPD data and phase evolution from Rietveld analysis are shown in Fig. 1. Rietveld fits for all key stages of the reaction and extracted crystallographic information are shown in Fig. S1, S2 and Tables S1, S2 in the ESI.† Both visual inspection and fitting of the data confirm a very similar phase evolution to that found for TiNiSn. Elemental Sn is completely lost from the NPD patterns at ∼230 °C alongside Ni_3_Sn_4_ formation, whilst elemental Ti remains present. The remaining Ni reacts slowly up to ∼795 °C, as evidenced by the increasing Ni content in Ni_3_Sn_4_ and by the formation of Ni_3_Sn_2_. Above 800 °C, the Ni–Sn binaries disappear from the diffraction patterns and TiNi_2_Sn, TiNiSn and Ti–Ni phases appear.

**Fig. 1:**
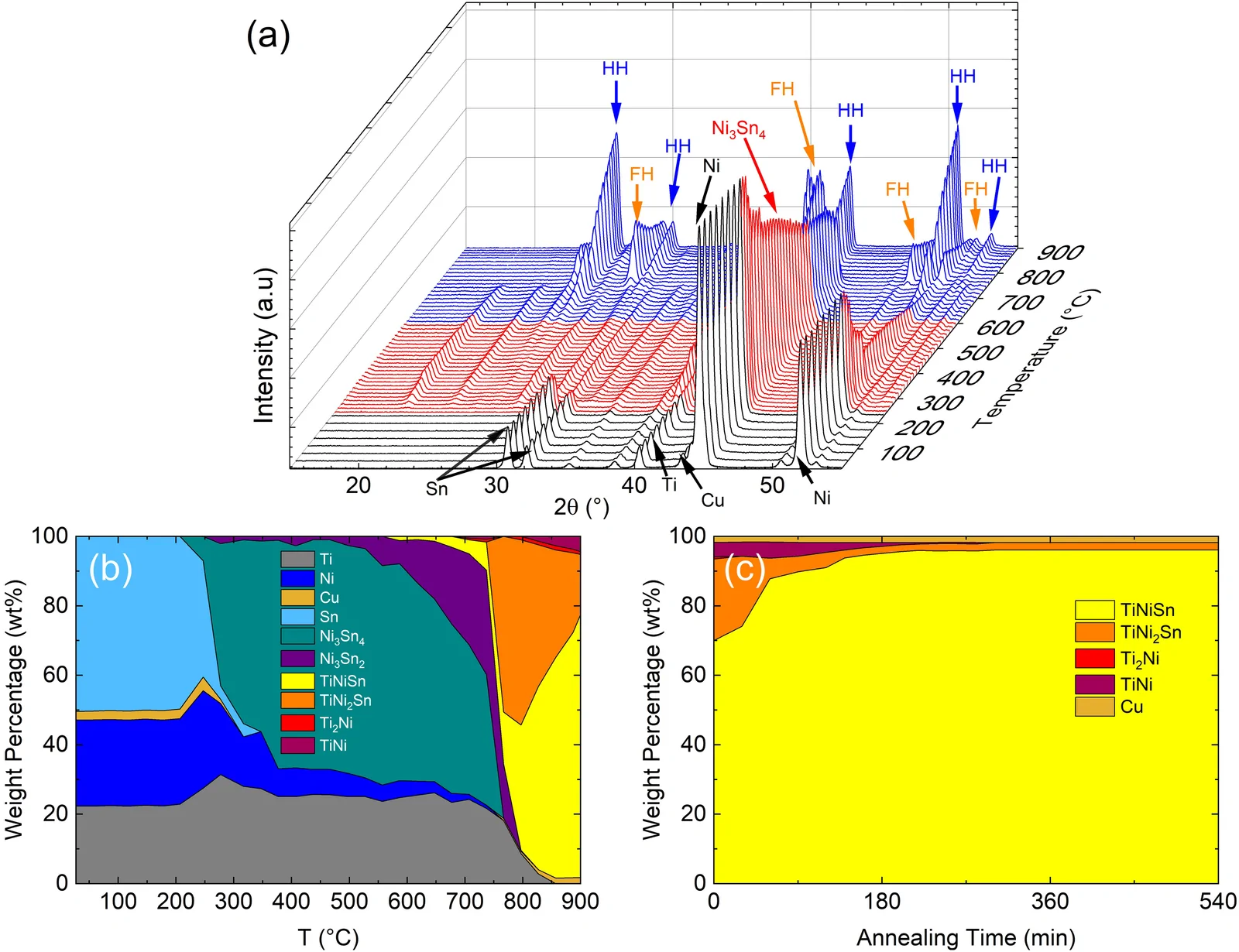
Phase evolution during synthesis of a TiNiCu_0.1_Sn sample from mixtures of elemental powders. Panel (a) shows a close-up of the NPD data (10 ≤ 2*θ* ≤ 55°) collected on heating (3 °C min^−1^) to 900 °C, with key phases indicated (FH = full-Heusler TiNi_2_Sn, HH = TiNiSn). The black datasets are where no reaction has yet occurred, the red datasets are after the melting of Sn and the blue datasets cover the onset of HH formation. Panel (b) shows the phase evolution during ramping to 900 °C extracted from Rietveld refinement and panel (c) shows the rapid conversion to the HH phase during heating at 900 °C.

The Cu dopant behaves unexpectedly during this process. In the first instance elemental Cu disappears when Sn melts, suggesting it gets incorporated into the Ni_3_Sn_4_ phase, as no crystalline Cu–Sn phases are observed. Another possible explanation is that Cu is incorporated in a low melting point phase. However, at this point in the reaction, there does not appear to be a large molten component, with all Sn in the reaction mixture accounted for by the crystalline phases (Fig. S3, ESI†). Furthermore, elemental Cu reappears after melting of the Ni–Sn phases, suggesting it was incorporated in Ni_3_Sn_4_. Upon the reappearance of Cu, when TiNi_2_Sn, TiNiSn and Ti–Ni form, Cu is also found in the HH phase, occupying the 4d site with an initial 0.03(1) occupancy.

As observed for TiNiSn without Cu, annealing at 900 °C is required to drive the reaction to completion. This involves the Ti–Ni binaries fully reacting out, reducing the amount of FH phase. No significant change in phase distribution is observed after 3–4 h, which is two times faster than for TiNiSn. This suggests the presence of a mineralising effect, linked to the presence of Cu, where reaction rates are increased by partial dissolution of reactants in molten phases. This can occur at interfaces and does not require large amounts of molten phases. During the 900 °C stage, part of the Cu is incorporated in the HH structure, with a final fitted 4d-site occupancy of 0.05(1) and part remains present as elemental Cu.

The NPD results demonstrate that high quality TiNiSn samples can be prepared in 8–9 h, including ramping up the furnace. However, most literature protocols use arc melting and employ much longer annealing times.^6^ Reducing furnace heating will enable substantial energy savings. Our results suggest that HH alloys can be made in a more energy efficient manner, without reverting to specialised routes such as microwave and self-propagating combustion synthesis.^13,14^

To explore possible faster synthesis, we prepared TiNiCu_0.03_Sn samples using a powder route mimicking the NPD experiment. An initial 5-gram batch was produced by heating cold-pressed disks of mixed powders for 24 h at 900 °C. Half of this sample was then annealed for another more typical 168 h period. The two samples were ball milled and hot pressed into dense disks for structural and TE characterisation. Full details of the sample processing are given in the ESI.†

Inspection of the XRD data showed good overall sample purity, for both samples, both before and after hot pressing (Fig. S4, ESI†). The results of Rietveld fits are summarised in Table S3 (ESI†). The 24 h sample has a small Sn impurity and shows a small increase in lattice parameter after hot pressing, consistent with a higher 4d site occupation. A similar increase in lattice parameter is seen in the 168 h sample. The fitted 4d site occupancies of these samples are 0.039(8) for the 24 h sample and 0.065(6) for the 168 h sample. This exceeds the nominal Cu content and signals the incorporation of Ni, as found in earlier work.^7^ The presence of excess Ni in the HH structure is supported by the observation of Ni_3_Sn_4_ (∼3 wt%) and Ni_3_Sn_2_ (∼2 wt%) impurities in the 168 h sample (Fig. S4, ESI†). As reported by Galazka *et al.*, these phases are the byproducts of the formation of Ti oxides.^15^ Oxidation of Ti would leave an excess of Ni and Sn in the sample, and some part of this excess Ni is incorporated on the 4d site. The density of the samples reduces from 99(1)% of theoretical for the 24 h sample to 95(1)% for the 168 h sample, which could be a consequence of HH grain fusion being compromised by surface oxides.

A comparison of the TE properties of the annealed samples is shown in Fig. 2. The 24 h sample has larger *S* values over the whole *T* range, indicative of a slightly lower doping level. Above 500 K, the 168 h sample shows a reduction in *S*(*T*). This is absent in the 24 h sample and indicates the onset of minority carrier conduction, which could be caused by carrier injection from the Ni–Sn impurities or by a reduced bandgap. A reduced bandgap due to in-gap states is a known consequence of the presence of Ni interstitials.^16^ Using the Goldsmid-Sharp equation, *E*_g_ = 2e*S*_max_*T*_max_, yields bandgap values of 0.28(1) eV and 0.22(1) eV for the 24 h and 168 h samples, consistent with the higher levels of interstitial metals in the 168 h sample from XRD.

**Fig. 2:**
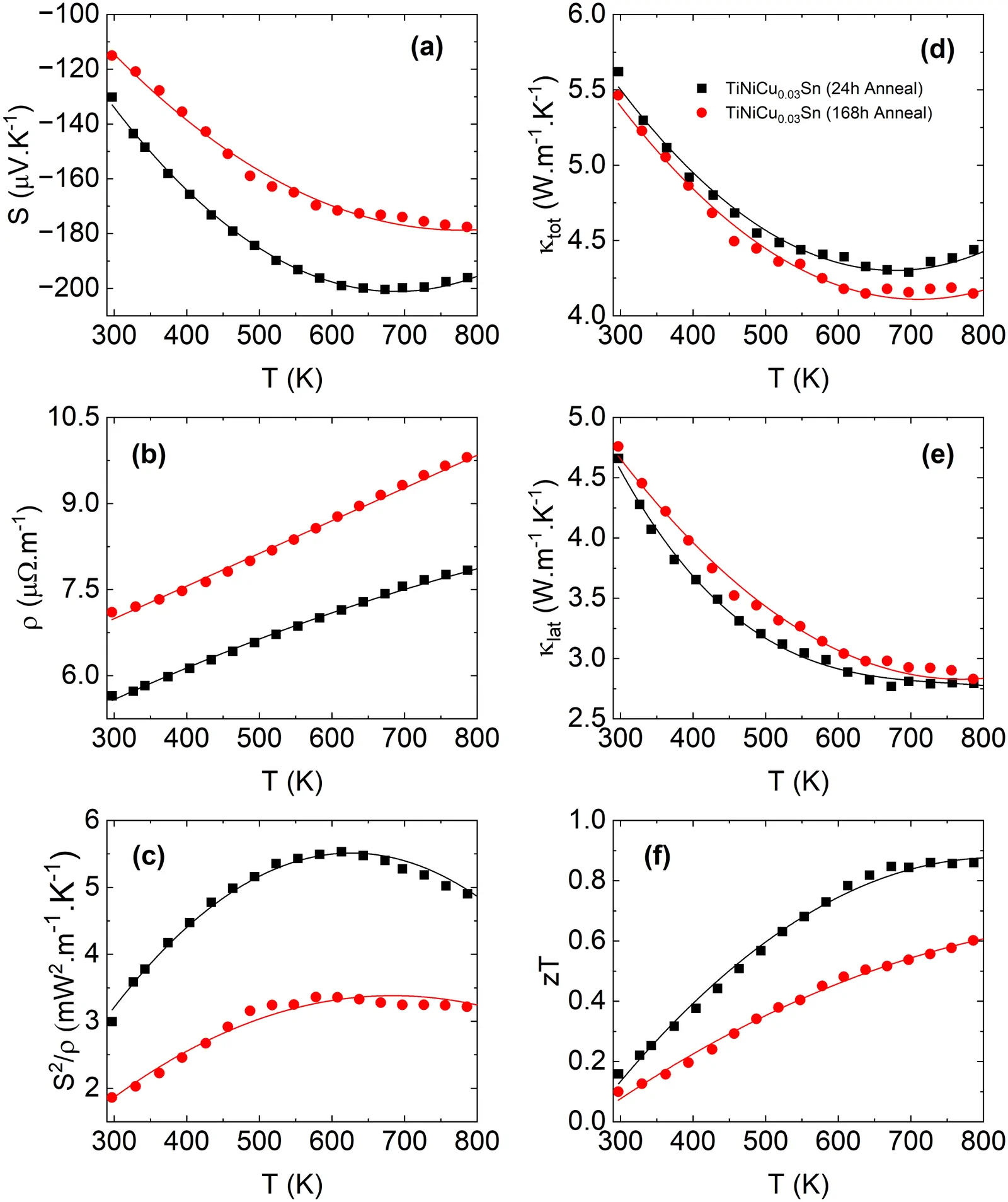
Temperature dependent thermoelectric properties for the 24 h and 168 h annealed TiNiCu_0.03_Sn samples. Panel (a) shows the Seebeck coefficient *S*, (b) the electrical resistivity *ρ*, (c) the power factor *S*^2^/*ρ*, (d) the total thermal conductivity *κ*, (e) the lattice thermal conductivity *κ*_L_, and (f) the figure of merit *zT*. Lines are guides to the eye.

The *κ*_L_(*T*) is near identical for both samples, with 323 K values of 4.7 W m^−1^ K^−1^, decreasing to 2.8 W m^−1^ K^−1^ at 793 K. These low values are consistent with the strong impact of interstitial metals, which have a comparable disorder effect to using heavy Hf.^7,8^ The total *κ* is substantially larger than *κ*_L_ with values of 5.5 W m^−1^ K^−1^ at 323 K, decreasing to 4.2–4.5 W m^−1^ K^−1^ at 793 K, which is due to the large *κ*_el_ of these materials (Fig. S5, ESI†).

The largest difference between the samples is observed in *ρ*(*T*) (Fig. 2b). The 24 h sample has 25% lower *ρ*(*T*) values across the whole *T* range. This occurs despite a lower doping level, indicating that the electron mobility in the 24 h sample is much higher. Together with the larger *S*(*T*), this explains the higher peak power factor, PF = *S*^2^/*ρ* = 5.5 mW m^−1^ K^−2^, compared to PF = 3.5 mW m^−1^ K^−2^ for the 168 h sample (Fig. 2c). The PF for the 24 h sample is amongst the highest values reported in the literature.^6,17^

The large PF and low *κ*_L_ for the 24 h sample support a high peak *zT* = 0.9 at 723 K. This performance is competitive with the best reported XNiSn materials prepared by melting routes (Fig. S6, ESI†),^6^ and matches our own results on arc-melted TiNiCu_0.03_Sn.^11^ This demonstrates that high performance can be achieved using a powder-based route. It also confirms that alloying with Zr/Hf may not be essential for applications. The 168 h sample with its compromised PF only reaches *zT* = 0.6, which is identical to the values we obtained in earlier work that also used long annealing.^7^ The stability of the samples under measurement conditions was confirmed using XRD (Fig. S7, ESI†).

The weighted mobility *μ*_w_,^18^ allows the electronic performance of materials to be compared, independent of doping level. The calculated *μ*_w_ is shown in Fig. S5 (ESI†), with 20–25% lower values over the whole *T* range for the 168 h annealed sample. The degraded performance is largely due to the increased *ρ*(*T*) of the 168 h sample.

A substantially different *ρ*(*T*) for compositionally similar samples is likely to be caused by extrinsic effects, including higher porosity and grain boundary (GB) resistances. Atom probe tomography has revealed the presence of mixed Ti–O/Cu/C GB complexions in TiNiCu_0.05_Sn samples prepared from powders and using long annealing.^19,20^ However, the impact of these GB phases on the TE properties is unclear. The degrading impact of insulating GB phases has been investigated in other HH materials, particularly NbFeSb, although here the cause is the depletion of Ti but not oxide formation.^21^

To probe the microstructure and oxide formation, scanning electron microscopy, electron backscattered diffraction (EBSD) and energy dispersive X-ray spectroscopy (EDX) analysis was undertaken (Fig. 3). The 24 h sample has no visible porosity, in agreement with its full density. By contrast, the 168 h sample has microscale pores throughout the 40 × 60 μm^2^ cross section, suggesting poor grain fusion. Furthermore, the 168 h sample has much smaller grain sizes (Fig. 3c). The grain size distribution has a stronger weighting towards grain sizes below 3 μm, with no grains in excess of 10 μm. The EDX maps in Fig. S8 (ESI†) confirm the homogeneity of the HH grains. However, Ti–O complexions are found at GBs in both samples, regardless of annealing time. The key difference is that after 24 h annealing, the oxidation is more localised, maintaining a larger average grain size and not preventing densification. By contrast, after 168 h annealing, the oxidation is more widespread, due to the increased time at reactive temperatures.^15^ This leads to more extensive Ti–O GB complexions, smaller grain sizes and increased GB density and compromised densification, all contributing towards the high *ρ*(*T*).

**Fig. 3:**
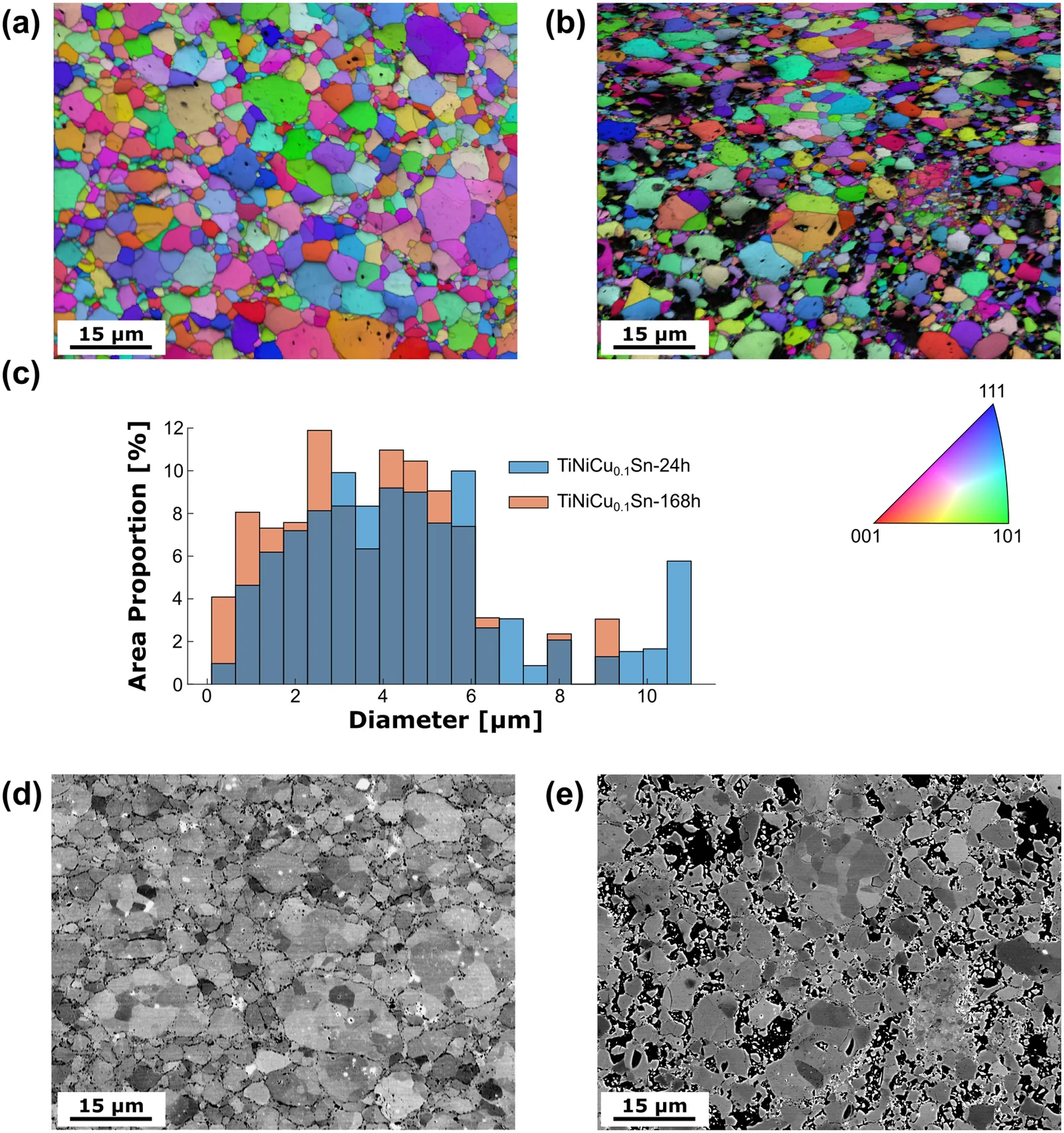
- Microscopy analysis of TiNiCu_0.03_Sn. EBSD maps for (a) the 24 h and (b) the 168 h annealed samples; (c) shows the average grain size distribution from the EBSD maps; BSE images of the (d) 24 h and (e) 168 h annealed samples, where greyscale contrast is used to highlight the grains.

To conclude, the main mechanistic insight is that Cu acts as a mineralizer during the annealing stage to afford faster HH phase formation, with Cu also being incorporated on the interstitial 4d position in the HH phase. Cu does not impact strongly on the initial sequence of phase formation, which is similar to that observed for TiNiSn without Cu. Long annealing leads to increasing Ti–O complexion phases at grain boundaries, reducing the average grain size and compromising sample densification, and resulting in increased sample resistances. Minimising oxidation is key for performance with a current highest *zT* = 0.9 at 723 K for TiNiCu_0.03_Sn annealed for 24 h.

BFK and JWGB acknowledge the EPRSC (EP/Y016459/1) and ILL for award of the beamtime.^22^ ABN acknowledges the EPRSC (EP/L017008/1, EP/R023751/1 and EP/T019298/1).

## Conflicts of interest

There are no conflicts of interest to declare.

## Supplementary Material

CC-061-D4CC06695B-s001

## Data Availability

The research data underpinning this publication can be accessed at https://doi.org/10.17630/3376dc09-fa5c-4c1d-822e-6d502c2a6ff0.

## References

[cit1] Hendricks T., Caillat T., Mori T. (2022). Energies.

[cit2] Yan Q. Y., Kanatzidis M. G. (2022). Nat. Mater..

[cit3] Snyder G. J., Toberer E. S. (2008). Nat. Mater..

[cit4] Quinn R. J., Bos J.-W. G. (2021). Mater. Adv..

[cit5] Zhu T., Fu C., Xie H., Liu Y., Zhao X. (2015). Adv. Energy Mater..

[cit6] Gürth M., Rogl G., Romaka V. V., Grytsiv A., Bauer E., Rogl P. (2016). Acta Mater..

[cit7] Barczak S. A., Halpin J. E., Buckman J., Decourt R., Pollet M., Smith R. I., MacLaren D. A., Bos J.-W. G. (2018). ACS Appl. Mater. Interfaces.

[cit8] Barczak S. A., Quinn R. J., Halpin J. E., Domosud K., Smith R. I., Baker A. R., Don E., Forbes I., Refson K., MacLaren D. A., Bos J.-W. G. (2019). J. Mater. Chem. A.

[cit9] Yan R., Shen C., Widenmeyer M., Luo T., Winkler R., Adabifiroozjaei E., Xie R., Yoon S., Suard E., Molina-Luna L., Zhang H., Xie W., Weidenkaff A. (2023). Mater. Today Phys..

[cit10] Sadia Y., Lumbroso D., Gelbstein Y. (2023). Materials.

[cit11] Quinn R. J., Go Y., Naden A. B., Bojtor A., Paráda G., Shawon A. K. M. A., Domosud K., Refson K., Zevalkink A., Neophytou N., Bos J.-W. G. (2025). Adv. Phys. Res..

[cit12] Barczak S. A., Kennedy B. F., da Silva I., Bos J.-W. G. (2023). Chem. Mater..

[cit13] Birkel C. S., Zeier W. G., Douglas J. E., Lettiere B. R., Mills C. E., Seward G., Birkel A., Snedaker M. L., Zhang Y., Snyder G. J., Pollock T. M., Seshadri R., Stucky G. D. (2012). Chem. Mater..

[cit14] Su X., Fu F., Yan Y., Zheng G., Liang T., Zhang Q., Cheng X., Yang D., Chi H., Tang X., Zhang Q., Uher C. (2014). Nat. Commun..

[cit15] Gałązka K., Populoh S., Sagarna L., Karvonen L., Xie W., Beni A., Schmutz P., Hulliger J., Weidenkaff A. (2014). Phys. Status Solidi (a).

[cit16] Schmitt J., Gibbs Z. M., Snyder G. J., Felser C. (2015). Mater. Horiz..

[cit17] Zhu H., Li W., Nozariasbmarz A., Liu N., Zhang Y., Priya S., Poudel B. (2023). Nat. Commun..

[cit18] Snyder G. J., Snyder A. H., Wood M., Gurunathan R., Snyder B. H., Niu C. (2020). Adv. Mater..

[cit19] Halpin J. E., Jenkins B., Moody M. P., Webster R. W. H., Bos J.-W. G., Bagot P. A. J., MacLaren D. A. (2022). ACS Appl. Electron. Mater..

[cit20] He H., Halpin J. E., Popuri S. R., Daly L., Bos J.-W. G., Moody M. P., MacLaren D. A., Bagot P. A. (2022). Microsc. Microanal..

[cit21] Bueno Villoro R., Zavanelli D., Jung C., Mattlat D. A., Hatami Naderloo R., Pérez N., Nielsch K., Snyder G. J., Scheu C., He R., Zhang S. (2023). Adv. Energy Mater..

[cit22] Bos J.-W. G. and Ritter C., Institut Laue-Langevin (ILL), 2023 10.5291/ILL-DATA.EASY-1132

