# Supplementary material for: Influence of Cu on the sustainable synthesis and thermoelectric properties of the half-Heusler TiNiSn
Source: Chem Commun (Camb). 2025 Apr 4;61(39):7117–20. doi: 10.1039/d4cc06695b (PMC13559778; doi:10.1039/d4cc06695b)
Supplement: Supplementary file 1 [file CC-061-D4CC06695B-s001.pdf]

Supplementary Information (SI) for ChemComm.
This journal is © The Royal Society of Chemistry 2025

ESI for

Influence of Cu on the Sustainable Synthesis and Thermoelectric Properties of the half-Heusler TiNiSn

Blair. F. Kennedy[a], Aaron B. Naden[a], Clemens Ritter[b], and Jan-Willem G. Bos[a*]

[a] *EaStCHEM School of Chemistry, University of St Andrews, North Haugh, St Andrews, KY16 9ST, UK.*

[b] *Institut Laue-Langevin, 71 Avenue des Martyrs, CS20156, 38042 Grenoble Cédex 9, France.*

Email: *j.w.g.bos@st-andrews.ac.uk*

Experimental:

To investigate the impact of Cu on the formation of TiNiSn, a sample with nominal $TiNiCu_{0.1}Sn$ composition was prepared. This high Cu content is near the solubility limit of Cu and was chosen to allow any impact of Cu to be easily followed by neutron powder diffraction (NPD). Optimal doping levels for thermoelectric applications are lower with arc melted $TiNiCu_{0.03}Sn$ reaching $zT = 0.9$ at 800 K.[1] Stochiometric amounts of Ti (Fisher Scientific, 99.999%, 325 mesh), Ni (Fisher Scientific, 99.999%, 100 mesh), Cu (Merck, 99.95 %, 100 mesh) and Sn powders (Fisher Scientific, 99.999%, 100 mesh) with a total mass of 5 gram were mixed using a mortar and pestle for 20 minutes in an Ar-filled glove box. The mixture was cold pressed at 10 tonnes under Ar atmosphere to form several 13 × 1.5 mm cylindrical disks. The disks were then cut into quarters using a hand saw. Stacks of the quartered pellets were loaded into a V can to a total height of ~5 cm.

NPD data on the stacked pellets was collected on the D20 diffractometer at the Institut Laue-Langevin (ILL), Grenoble, France. The V can with the stacked pellets was placed into a furnace and heated to 900 °C with a heating rate of 3 °C/min. Datasets were collected between $20 \leq 2\theta \leq 140°$ over 10-minute intervals throughout the experiment. During the ramping stage this corresponds to a ~30 °C increase in temperature. After reaching 900 °C, the sample was held for 540 minutes. Temperature readings were taken from a thermocouple ~5 mm away from the top of the vanadium can.

For assessing the impact of annealing on thermoelectric performance, one 5-gram $TiNiCu_{0.03}Sn$ sample was prepared using the same approach as for the NPD experiment. All sample handling was done under protective Ar gas environment. Stoichiometric amounts of elemental powders were mixed using mortar and pestle, cold pressed at 10 tonnes into 13 mm

disks, which were wrapped in Ta foil and heated in an evacuated quartz ampoule in a muffle furnace. The heating rate was 3 °C/min and the sample was kept at 900 °C for 24 hours. Towards the end of the heating stage, the sample was quenched from the furnace. At this point half of the pellet was reground, cold-pressed at 10 tonnes and annealed for a further 168 hours at 900 °C. The resulting two ~2.5-gram samples (one with 24h, the other with 24h+168h heating) were pulverized under Ar atmosphere using a MM400 Retsch shaker mill. This used 15 mL stainless steel cups, a single 10 mm diameter ball, a frequency of 20 Hz for 60 minutes total time, with 10 minutes rest periods after every 10 minutes of milling. The milled powders were hot-pressed using a homebuilt instrument. Dense disks were obtained using a temperature of 900 °C and applied pressure of ~80 MPa.

X-ray Powder Diffraction (XRD) data on the $TiNiCu_{0.03}Sn$ samples was collected on a Malvern Panalytical diffractometer in a Bragg Brentano setup using monochromated Cu radiation. Data was collected on the ball milled powder after annealing of the cold pressed disks, and on the dense disks after hot pressing. All Rietveld fitting was carried out using the General Structure Analysis System II (GSAS-II) suite of programs.[2]

Electron backscatter diffraction (EBSD) was performed with simultaneous energy dispersive X-ray spectroscopy (EDX) on an FEI Scios equipped with an EDAX Hikari EBSD camera and an EDAX Octane plus EDX spectrometer. Samples were prepared by embedding in epoxy resin (Struers Epofix) followed by grinding & polishing with SiC paper (600 and 1200P), polycrystalline diamond (6, 3 and 1 μm) and colloidal silica.

The electrical resistivity (ρ) and Seebeck coefficient (S) were measured using a Linseis LSR-3 instrument on bar-shaped specimens (~1.5 × 2 × 10 mm^3) under a static He atmosphere. All bars were cycled twice between 300-773 K, with no degradation of performance observed. XRD collected after LSR measurements (Fig. S6) showed no evidence of thermal degradation. The thermal diffusivity (α) was measured using a Linseis LFA-1000 laser flash instrument on hot-pressed disks of ~13 mm diameter and ~1.5 mm thickness, under dynamic vacuum conditions. The thermal conductivity κ was then calculated from the equation $\kappa = \alpha \times C_p \times d$, with C_p the Dulong Petit specific heat and d the density obtained using the Archimedes method (Table S3). The electronic thermal conductivity $\kappa_{el} = LT/\rho$ was estimated using the Wiedemann-Franz law, using experimental S to calculate the Lorenz number (L).[3] The lattice thermal conductivity κ_L was calculated using $\kappa_L = \kappa - \kappa_{el}$. The weighted mobility, μ_w was calculated from the experimental S and ρ data following literature procedures.[4]

Table S1 - Weight percentage (wt.%) and composition of phases identified from D20 NPD data during the heating up from 30 – 900 °C to form the target $TiNiCu_{0.1}Sn$ alloy. The given temperature is the average value over the data collection time.

T (°C)	Ti	Ni	Cu	Sn	$Ni_{3+x}Sn_4$		$Ni_{2+2z}Sn_2$		$TiNiCu_ySn$		$TiNi_2Sn$	Ti_2Ni	TiNi
	wt%	wt%	wt%	wt%	wt%	x	wt%	z	wt%	y	wt%	wt%	wt%
30	22.3(3)	24.8(4)	2.5(1)	50.4(3)	0	0	0	0	0	0	0	0	0
60	22.3(4)	24.9(2)	2.5(1)	50.3(3)	0	0	0	0	0	0	0	0	0
90	22.5(4)	24.8(2)	2.6(1)	50.1(4)	0	0	0	0	0	0	0	0	0
120	22.3(4)	24.8(2)	2.6(1)	50.3(4)	0	0	0	0	0	0	0	0	0
150	22.6(4)	24.8(2)	2.6(1)	50(4)	0	0	0	0	0	0	0	0	0
180	22.4(4)	24.7(2)	2.7(1)	50.2(4)	0	0	0	0	0	0	0	0	0
210	22.9(3)	24.6(2)	2.8(1)	49.7(4)	0	0	0	0	0	0	0	0	0
240	27.5(4)	28.1(2)	3.9(1)	33.5(4)	7.0 (2)	0 (f)	0	0	0	0	0	0	0
270	31.4(3)	20.5(2)	1.2(1)	3.9(2)	40.9(3)	0 (f)	2.1(1)	0.5 (f)	0	0	0	0	0
300	28(2)	14.3(1)	0	3.8(2)	52.9(3)	0.04(1)	1.0(1)	0.5 (f)	0	0	0	0	0
330	27.4(2)	16.4(1)	0	0	54.9(3)	0.06(1)	1.3(1)	0.5 (f)	0	0	0	0	0
360	25.1(3)	8.0(1)	0	0	65.8(3)	0.04(1)	1.1(1)	0.5 (f)	0	0	0	0	0
390	25.1(2)	8.3(1)	0	0	63.9(2)	0.07(1)	2.7(1)	0.5 (f)	0	0	0	0	0
420	22.7(3)	7.2(1)	0	0	67.2(3)	0.09(1)	2.5(1)	0.5 (f)	0	0	0	0	0
450	21.7(2)	6.1 (1)	0	0	72.0(2)	0.15(1)	2.1 (1)	0.5 (f)	0	0	0	0	0
480	24.4(3)	6.7(1)	0	0	66.4(3)	0.14(1)	2.6(1)	0.5 (f)	0	0	0	0	0
510	25.1(4)	5.4(1)	0	0	66.0(4)	0.17(1)	3.5(1)	0.28(4)	0	0	0	0	0
540	23.7(4)	4.7(1)	0	0	63.2(6)	0.21(1)	8.4(4)	0.31(2)	0	0	0	0	0
570	24.8(3)	4.9(1)	0	0	62.4(4)	0.32(1)	6.7(2)	0.33(2)	1.2(1)	0 (f)	0	0	0
600	25.5(5)	4.0(1)	0	0	57.2(6)	0.25(1)	12.4(3)	0.42(2)	0.9(1)	0 (f)	0	0	0
630	26.2(5)	3.2(1)	0	0	52.6(6)	0.27(1)	16.6(5)	0.40(1)	1.4(1)	0 (f)	0	0	0
660	23.4(5)	2.6(1)	0	0	48.8(6)	0.39(1)	22.1(5)	0.48(1)	3.1(1)	0 (f)	0	0	0
690	24.3(6)	1.4(1)	0	0	43.1(7)	0.44(1)	26.2(6)	0.61(2)	4.0(1)	0 (f)	0	0.5(1)	0.5(1)
720	21.7(4)	0.9(1)	0	0	37.6(6)	0.50(1)	30.1(4)	0.46(2)	8.1(1)	0.03(1)	0	0.8(1)	0.8(1)
750	18.2(5)	0	0.6(1)	0	0	0	15.6(3)	0.41(1)	15.0(2)	0.03(1)	50.5(2)	0	0
780	8.7(4)	0	0.6(1)	0	0	0	0.3(1)	0.5 (f)	36.1(3)	0.03(1)	53.5(3)	1(1)	0
810	2.8(3)	0	1.2(1)	0	0	0	0	0	52.8(2)	0.03(1)	40.8(2)	1(1)	1.4(5)
840	0	0	1.6(1)	0	0	0	0	0	63.6(3)	0.03(1)	30.9(2)	1.2(4)	2.8(4)
870	0	0	1.7(2)	0	0	0	0	0	70.6(3)	0.03(1)	23.0(2)	1(1)	3.8(4)
900	0	0	1.8(2)	0	0	0	0	0	75.7(3)	0.04(1)	17.3(2)	0.8(4)	4.6(4)

Table S2 - Weight percentage (wt.%) and composition of phases identified from D20 NPD data during annealing at 900 ˚C to form the target $TiNiCu_{0.1}Sn$ alloy.

Time (Minutes)	$TiNiCu_ySn$*		$TiNi_2Sn$	Ti_2Ni	TiNi	Cu
	wt.%	y	wt.%	wt.%	wt.%	wt.%
0	70.0 (3)	0.04(1)	23.5(3)	0.6(1)	4.2(1)	1.7(1)
30	74.1(3)	0.05(1)	20.2(3)	0	4.1	1.6(2)
60	87.8(2)	0.05(1)	5.9(2)	0	4.7	1.6(2)
90	89.8(2)	0.05(1)	4.5(2)	0	4	1.7(1)
120	91(2)	0.05(1)	4.4(2)	0	2.8	1.8(1)
140	93.8(2)	0.05(1)	2.3(1)	0	2.1	1.8(2)
160	94.6(2)	0.05(1)	2.1(1)	0	1.5	1.8(2)
180	95.1(2)	0.05(1)	2(1)	0	1.1	1.8(2)
200	95.6(2)	0.05(1)	2(1)	0	0.6(1)	1.8(2)
220	96(2)	0.05(1)	1.8(1)	0	0.4(1)	1.8(2)
240	95.8(2)	0.05(1)	2.1(1)	0	0.4(1)	1.7(2)
260	95.9(2)	0.05(1)	2.0(1)	0	0.4(1)	1.7(2)
280	95.8(2)	0.05(1)	2.0(1)	0	0.5(1)	1.7(2)
300	96.1(2)	0.05(1)	2.1(1)	0	0	1.8(2)
320	96.1(2)	0.05(1)	2.1(1)	0	0	1.8(2)
340	96.1(2)	0.05(1)	2.1(1)	0	0	1.8(2)
400	96.1(2)	0.05(1)	2.1(1)	0	0	1.8(2)
450	96.1(2)	0.05(1)	2.1(1)	0	0	1.8(2)
500	96.1(2)	0.05(1)	2.1(1)	0	0	1.8(2)
540	96.1(2)	0.05(1)	2.1(1)	0	0	1.8(2)

*All HH phases have a fixed amount of Ni = 0.02 placed on the 4d site, alongside Cu, following earlier results.[5, 6]

Table S3 – Fitted lattice parameters for $TiNiCu_{0.03}Sn$ at key stages of sample synthesis and processing. Sample densities were obtained using the Archimedes method, % density is compared to the crystallographic density. (f = fixed).

Reaction Stage	a (Å)	Y2 Site Occupancy	Disk Density (g/cm^3)	Disk Density (%)
24h Anneal	5.9325 (1)	0 (f)	---	---
168h Anneal	5.9353(1)	0.01(1)	---	---
24h Anneal + Hot Press	5.9368(1)	0.039(8)	7.18(3)	100%
168h Anneal + Hot Press	5.9364(1)	0.065(6)	6.86(3)	95%

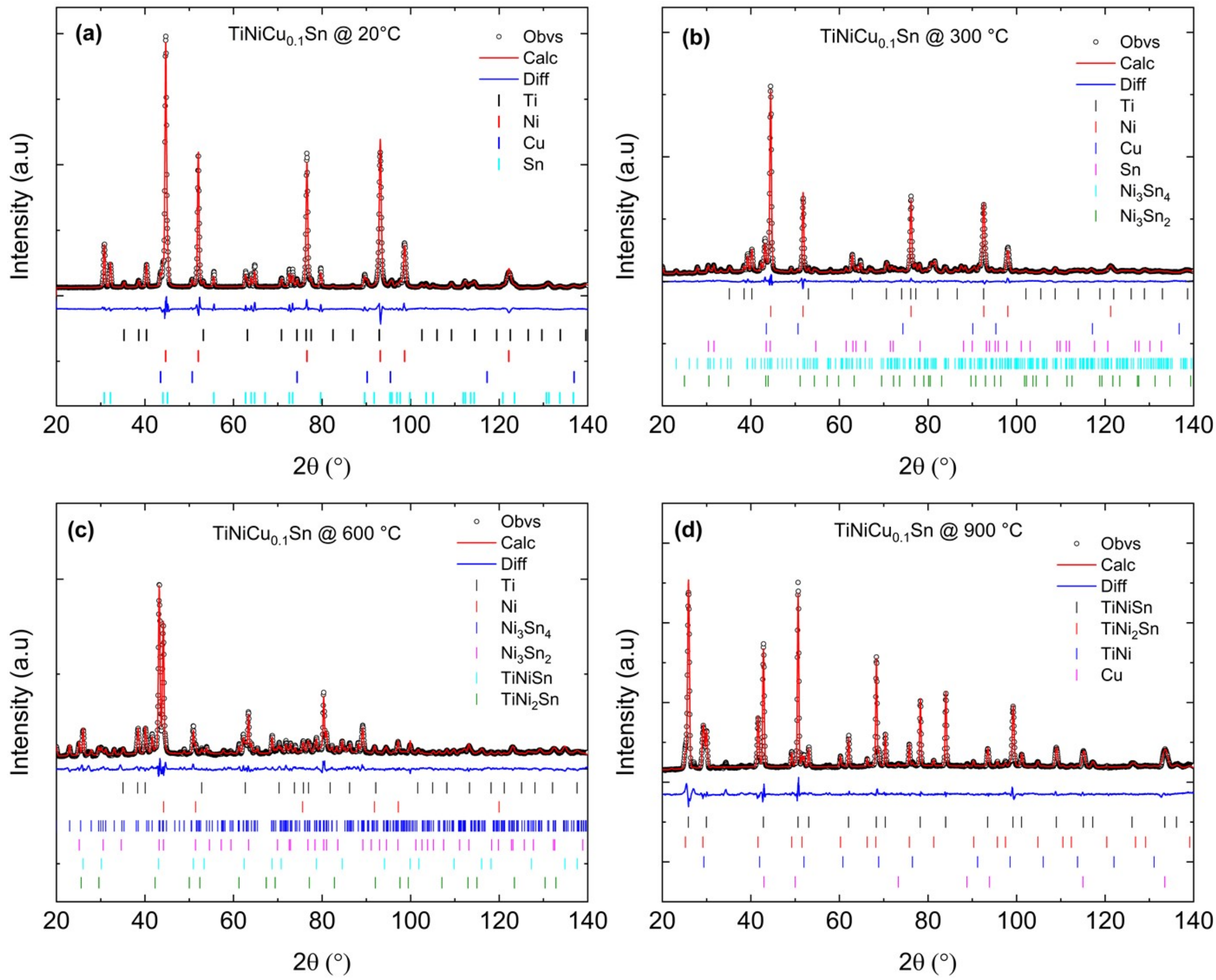

Figure S1 - Rietveld fits to D20 neutron powder diffraction data collected during ramping of $TiNiCu_{0.1}Sn$ to 900 °C. Panels show data at (a) 20 °C – showing the presence of Ti, Ni, Cu and Sn, (b) 300 °C – formation of Ni_3Sn_4 after melting of Sn between 230-320 °C, (c) 600 °C – with initial formation of $TiNiCu_ySn$ and (d) on reaching 900 °C – with the half-Heusler phase now the major phase, but in the presence of substantial secondary phases, including the reappearance of Cu.

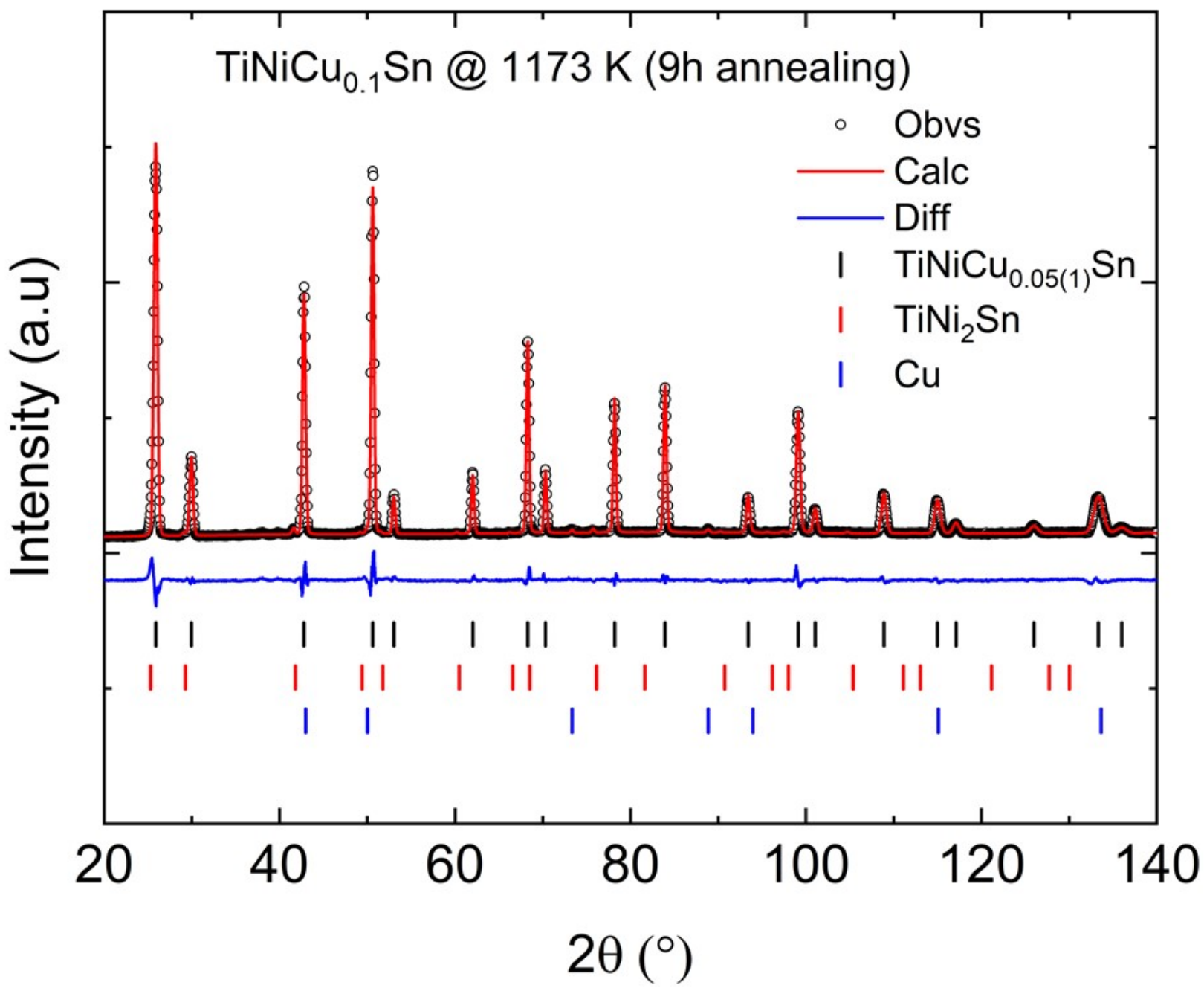

Figure S2 - Rietveld fit to D20 neutron powder diffraction data collected on $TiNiCu_{0.1}Sn$ after annealing at 900 °C for 540 minutes.

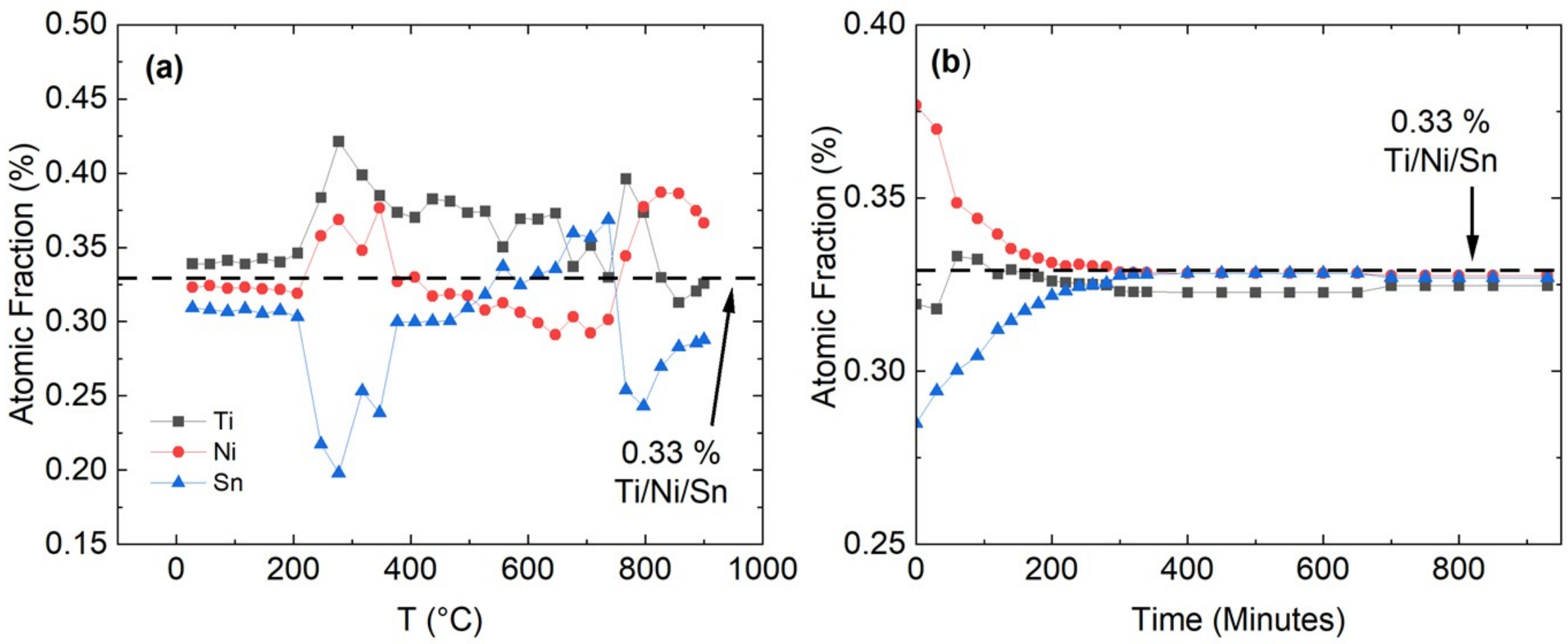

Figure S3 - Temperature evolution of the atomic fractions of Ti, Ni and Sn for the $TiNiCu_{0.1}Sn$ sample from Rietveld analysis of D20 NPD data. (a) shows the evolution during ramping at 3 °C/min to 900 °C. (b) shows the evolution during annealing at 900 °C and on cooling. Deviations from the nominal fractions (dashed line) signal the presence of molten phases, not accounted for in the Rietveld phase analysis. Cu has been excluded due to its low concentration.

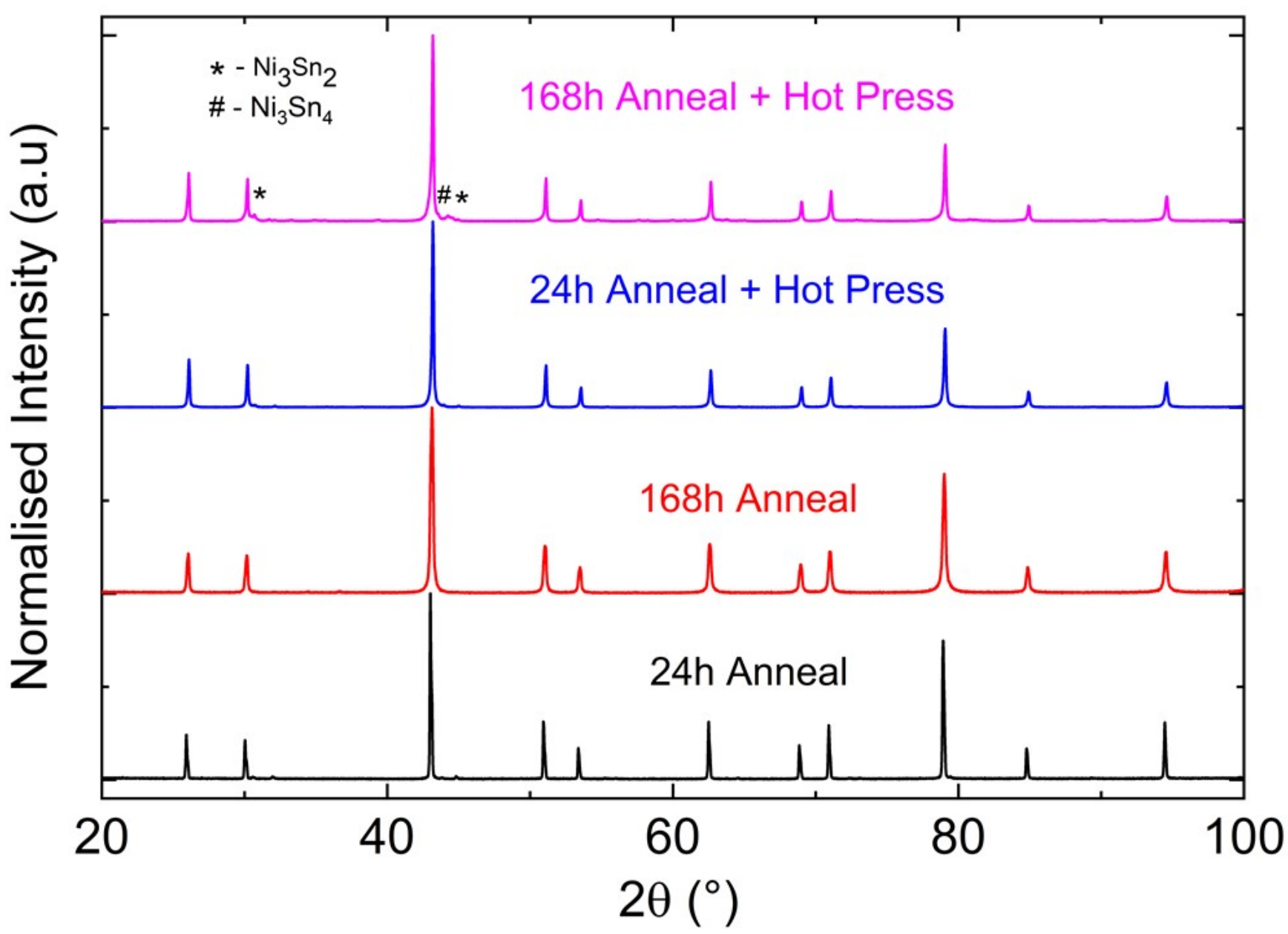

Figure S4 - Laboratory X-ray powder diffraction patterns of $TiNiCu_{0.03}Sn$, taken at key sample preparation stages.

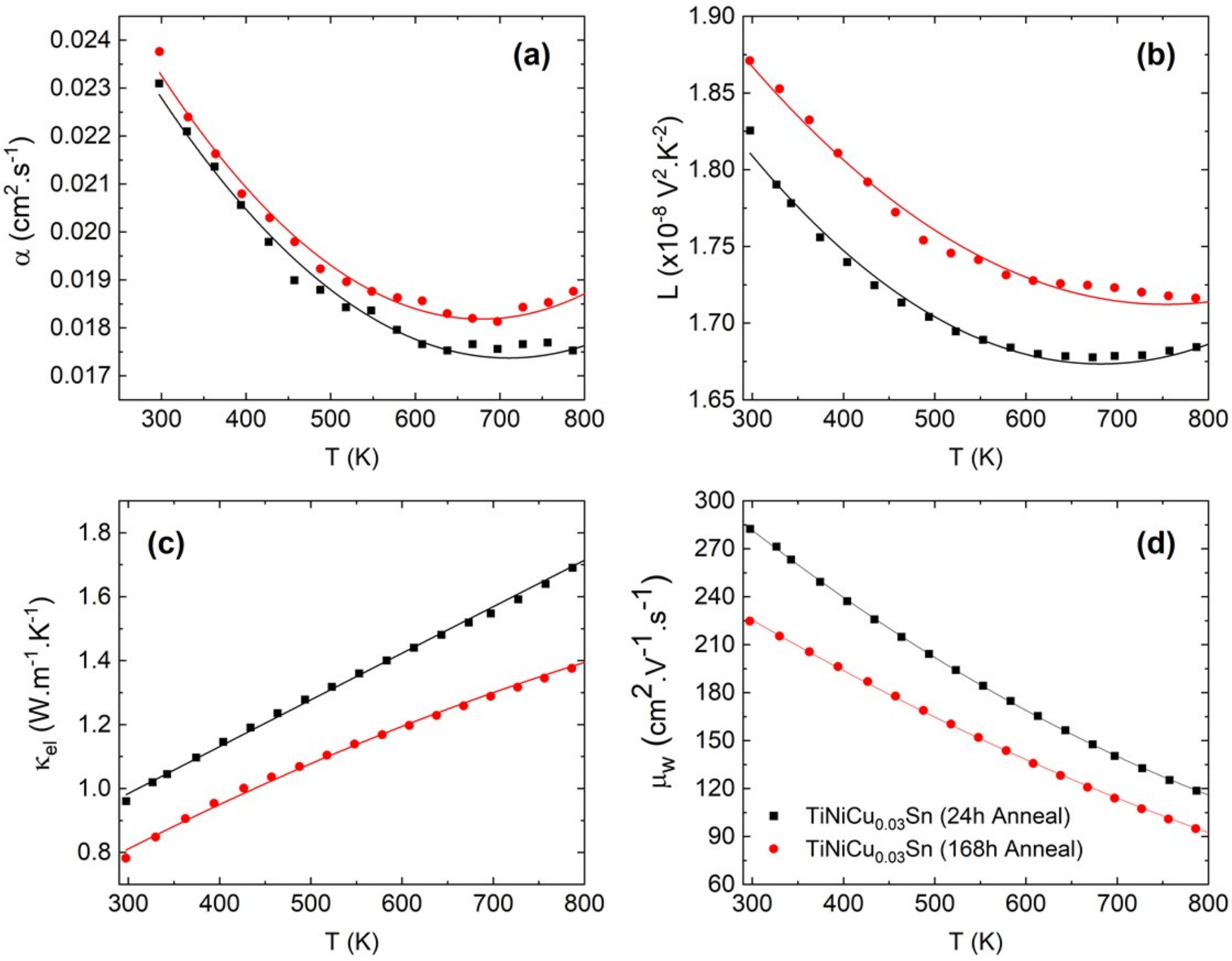

Figure S5 - Additional thermoelectric data for the $TiNiCu_{0.03}Sn$ samples. Panels show the temperature dependence of (a) the thermal diffusivity (α), (b) the Lorenz Number (L),[3] (c) $\kappa_{el} = LT/\rho$ and (d) the calculated weighted mobility μ_w.[4]

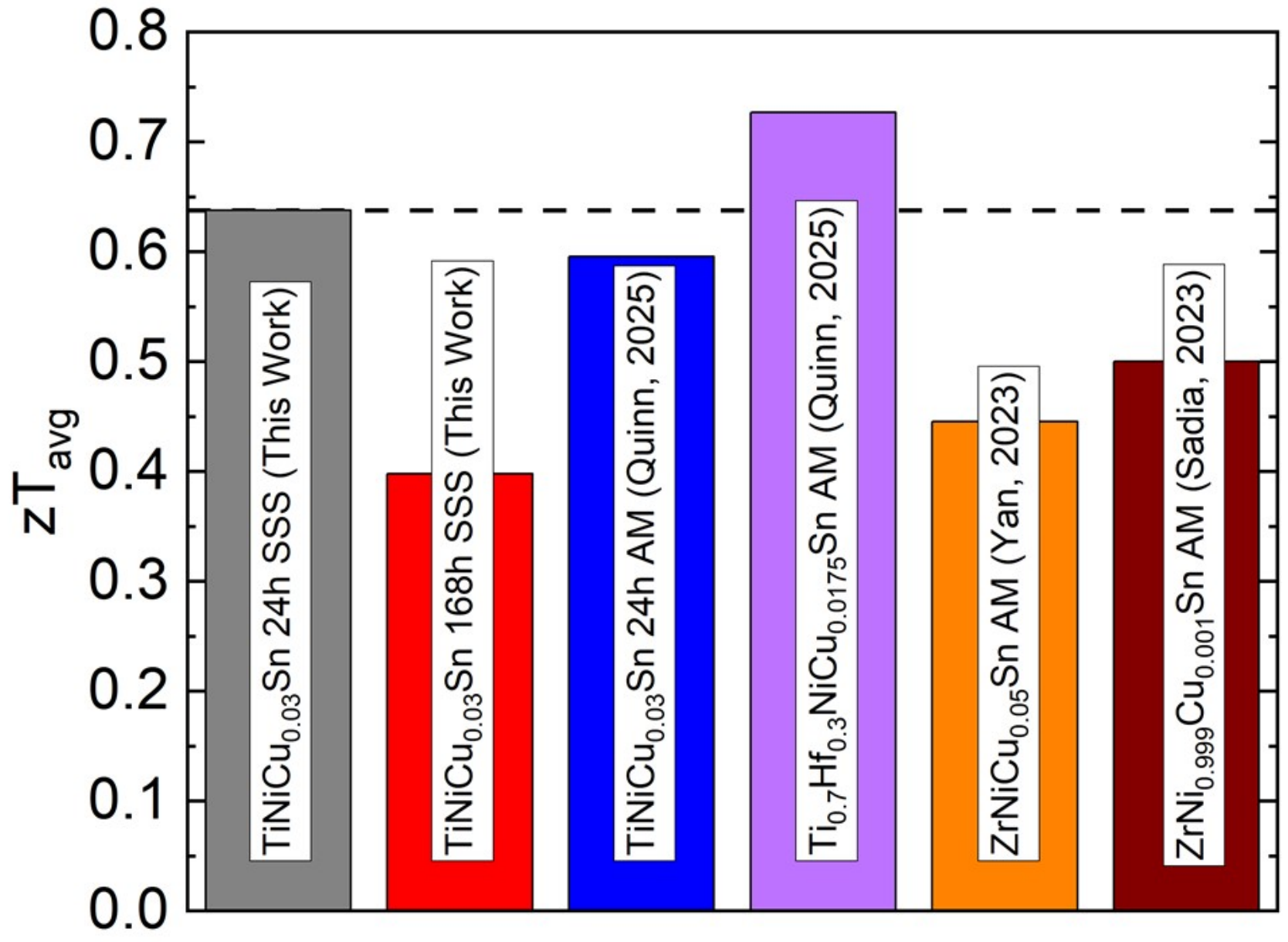

Figure S6 - Comparison of temperature-averaged *zT* values of the $TiNiCu_{0.03}Sn$ samples in this manuscript to values for the best performing Cu doped XNiSn materials from the literature. Temperature range 329-793 K. AM is prepared via arc melting. Literature data taken from Quinn et al.[1], Yan et al.[7] and Sadia et al.[8]

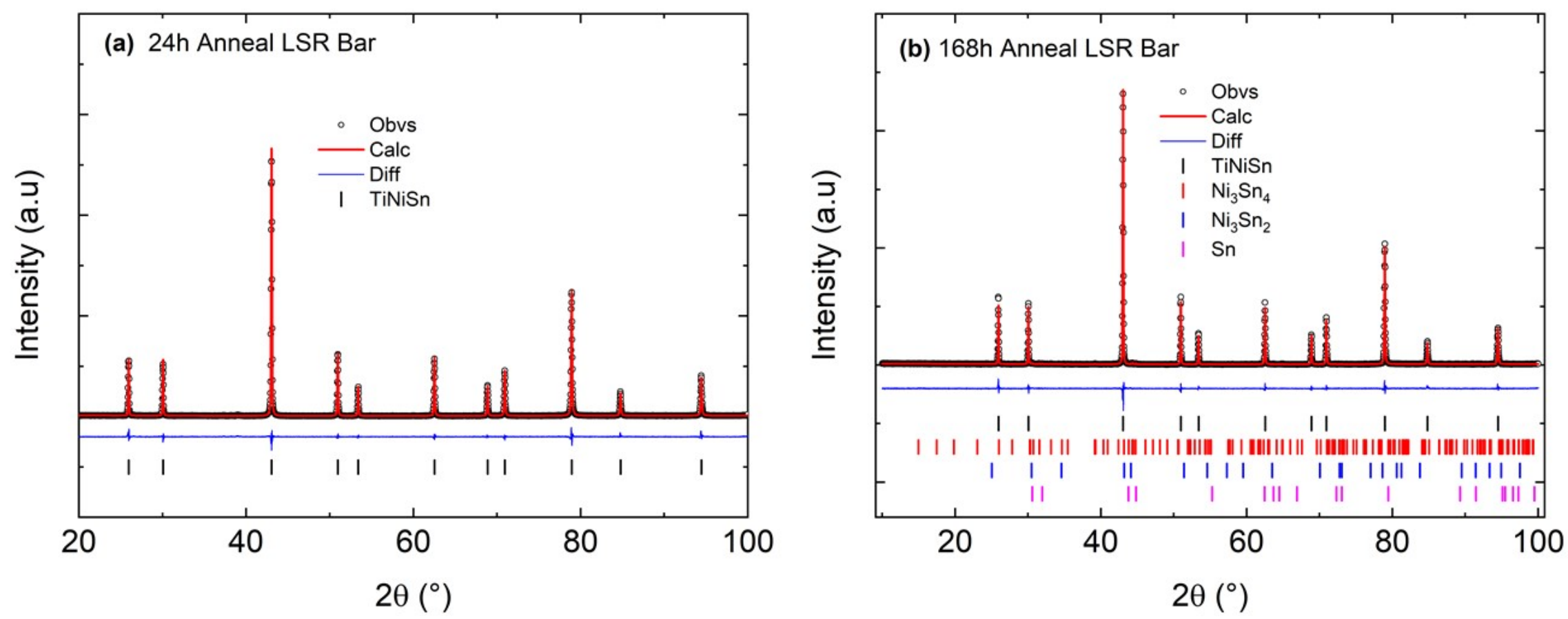

Figure S7 - Rietveld analysis of the laboratory XRD data collected on the $TiNiCu_{0.03}Sn$ bars after LSR measurement, illustrating unchanged phase composition compared to the samples before measurements. (a) shows the 24h annealed sample and (b) the 168h annealed sample.

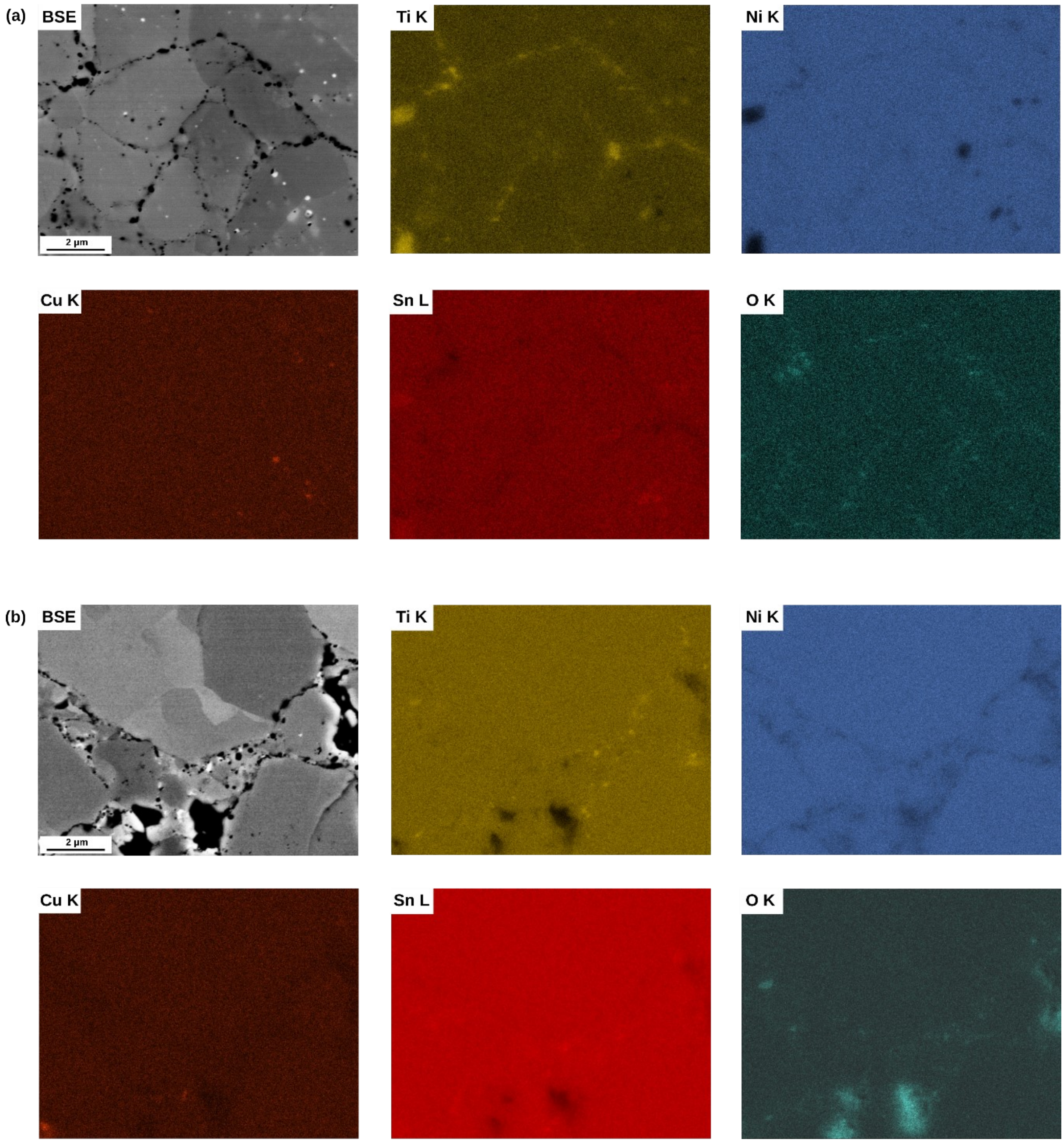

Figure S8 - Backscattered secondary electron (BSE) and Energy Dispersive X-ray Spectroscopy (EDS) SEM maps for $TiNiCu_{0.03}Sn$, highlighting the formation of Ti-O between half-Heusler grains and the otherwise homogenous distribution of Ti/Ni/Cu and Sn. Panel (a) is for the 24h annealed sample and (b) is for the 168h annealed sample.

References

1. R. J. Quinn, Y. Go, A. B. Naden, A. Bojtor, G. Paráda, A. K. M. A. Shawon, K. Domosud, K. Refson, A. Zevalkink, N. Neophytou and J.-W. G. Bos, *Advanced Physics Research*, 2025, DOI: https://doi.org/10.1002/apxr.202400179.
2. B. H. Toby and R. B. Von Dreele, *Journal of Applied Crystallography*, 2013, **46**, 544-549.
3. H.-S. Kim, Z. M. Gibbs, Y. Tang, H. Wang and G. J. Snyder, *APL Materials*, 2015, **3**, 041506.
4. G. J. Snyder, A. H. Snyder, M. Wood, R. Gurunathan, B. H. Snyder and C. Niu, *Advanced Materials*, 2020, **32**, 2001537.
5. S. A. Barczak, J. E. Halpin, J. Buckman, R. Decourt, M. Pollet, R. I. Smith, D. A. MacLaren and J.-W. G. Bos, *ACS Applied Materials & Interfaces*, 2018, **10**, 4786-4793.
6. R. A. Downie, R. I. Smith, D. A. MacLaren and J.-W. G. Bos, *Chemistry of Materials*, 2015, **27**, 2449-2459.
7. R. Yan, C. Shen, M. Widenmeyer, T. Luo, R. Winkler, E. Adabifiroozjaei, R. Xie, S. Yoon, E. Suard, L. Molina-Luna, H. Zhang, W. Xie and A. Weidenkaff, *Materials Today Physics*, 2023, **33**, 101049.
8. Y. Sadia, D. Lumbroso and Y. Gelbstein, *Materials*, 2023, **16**, 1902.
